# Relationship between implant stability on the abutment and platform level by means of resonance frequency analysis: A cross-sectional study

**DOI:** 10.1371/journal.pone.0181873

**Published:** 2017-07-24

**Authors:** Frederico Santos Lages, Dhelfeson Willya Douglas-de-Oliveira, Guilherme Siqueira Ibelli, Fatimah Assaf, Thallita Pereira Queiroz, Fernando Oliveira Costa

**Affiliations:** 1 Department of Periodontology, Federal University of Minas Gerais, Belo Horizonte, Minas Gerais, Brazil; 2 Department of Implantology, University Center of Araraquara, Araraquara, São Paulo, Brazil; Universita degli Studi di Roma La Sapienza, ITALY

## Abstract

Resonance frequency analysis (RFA) has become the main tool used to assess the osseointegration of dental implants. The objective of this study was to verify the relationship between the ISQ values with different prosthetic abutments and with the implant platform. The hypothesis was that ISQ values changes according to the abutment height. Twelve patients were included, whose contribution to the study was 31 dental implants (external hexagon connection implants, 4.1x10 mm). The temporary implant-supported crown and prosthetic components were removed and the following smartpegs were inserted, one at a time: type 1, in the implant platform (G1); type A3, in the microunit component with 1mm transmucosal height (G2) and type A3, in the microunit component with 5mm transmucosal height (G3). In all the smartpegs, RFA measurements were taken on mesial, distal, buccal and lingual surfaces. All evaluations were performed by a single calibrated examiner (ICC = 0.989). Data were analyzed by Friedman and Spearman correlation tests and log-linear marginal regression (p<0.05). The mean age of participants was 52.83 (± 3.77) years. There was statistically significant difference (p<0.001) among the mean ISQ of G1 (88.27 ±5.70); G2 (72.75 ±4.73) and G3 (66.33 ±3.67). There was statistically significant negative correlation between the ISQ and the measurement distance (rs:-0.852; p<0.001; R2:0.553). Measurement distance was significantly associated (p<0.001) with ISQ value in the log-linear regression. The abutment height has a significant impact on resonance frequency analysis measurements. The higher the transmucosal abutment height, the lower the implant stability quotient value. Clinically, the ISQ measured on the abutment cannot be compared with values measured on the implant platform.

## Introduction

In clinical research in implantology, there is great difficulty in assessing the osseointegration of implants. Nowadays, resonance frequency analysis (RFA) has become the main tool used, as it is a simple and noninvasive method that makes it possible to monitor implant stability throughout the required period [1].

RFA is measured by the Osstell^®^ device. This device that uses transducers connected to the implant or prosthetic components is available for various systems. The transducers (smartpegs) print a lateral force on the fixed components and the system shift is then measured. Thus, RFA measures the stiffness and deflection of the implant-bone complex [2]. The value obtained by Osstell^®^ is automatically translated into an index called the Implant stability quotient (ISQ), ranging from 1 to 100 (with 100 being the highest stability), and also allows stability to be evaluated over time, and to identify the conditions of bone around the implant [3,4]. In clinical practice, the implant stability can mainly be tested indirectly. However, caution should be exercised when judging implant systems exclusively on the basis of RFA and torque measurement [5].

The Osstell^®^ can be used at the time of implant insertion, during the healing period, and with the prosthesis in function [6].

Several clinical studies have demonstrated the reliability of this method for determining when to start loading on the implant [7–11], and the value of ISQ 70 is considered the threshold for insertion of the prosthesis [12].

However, in the literature, relationship between implant stability and success values have been observed only in measurements made directly on the implant platform, although the manufacturer of Osstell^®^ has indicated specific types of transducers to be applied to the prosthetic component (abutment). Nevertheless, there is no scientific evidence that elucidates the questions about this topic and allows comparisons to be made between these values.

Furthermore, it has been evident that after receiving the tightening torque, the prosthetic components must be held in position, and their removal must be prevented, because this may cause damage to both the threads of the implant and of the components themselves.

Considering the several clinical studies that have used resonance frequency analysis by means of Osstell^®^ to evaluate the stability of implants, and the absence of articles in the literature correlating the values obtained in the implant and abutment, the proposed study is a fundamental step towards facilitating the standardization of measurements and interpretation of the results obtained in each abutment.

The objective of this study was to verify the relationship between the ISQ values with different prosthetic abutments and with the implant platform. The hypothesis investigated was whether ISQ values changes according to abutment height and implant platform, at the same dental implant.

## Material and methods

### Sample size

This study was approved by the Ethics Committee on Human Research of Federal University of Minas Gerais under protocol 57216016.6.0000.5149. It was conducted in accordance with Declaration of Helsinki of 1975, revised in 2013. The participants signed a term of Free and Informed Consent before the study.

To determine the sample size, the calculation for correlation coefficient was used. The standard deviation (0.2) was obtained from a previous study using RFA in clinical trial [13]. The calculation, considering a 95% significance level and 80% power, determined that a minimum of 25 implants would be sufficient to detect a difference of 5 units in the implant stability quotient (ISQ) between groups.

All evaluations were performed by a single examiner (FSL). This researcher was trained and calibrated by the test-retest method, and the intraclass coefficient correlation was 0.989.

The patients were selected randomly among those who participated in a previous study [14], between February and April 2016. The inclusion criteria were: patients who were unilaterally or bilaterally edentulous in posterior mandibular area, with external hexagon connection implants, 4.1 mm in diameter (regular platform) and an implant length greater than or equal to 10mm (conventional implants). All patients eligible for selection had to have undergone the second surgical procedure and had to be rehabilitated with fixed provisional screw-retained implants. Patients who had mucositis, peri-implantitis and/or signs of bruxism were excluded from the sample.

Patients who met the inclusion criteria were required to sign a term of free and informed consent, and at the end of the treatment would be rehabilitated with metal-ceramic prosthesis on these implants.

### RFA evaluation

**Fig 1:**
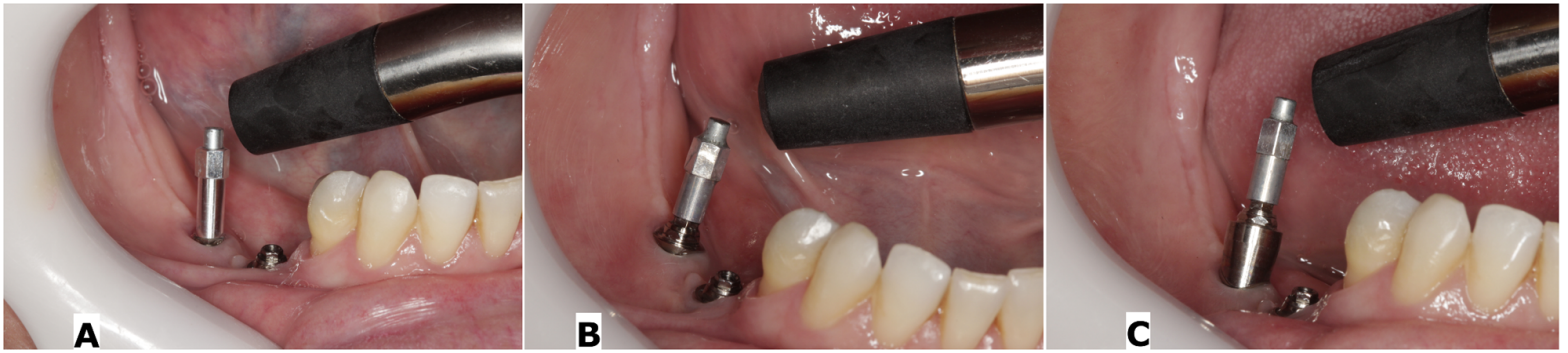
Resonance frequency analysis by Osstell. A) Implant platform B) 1mm microunit C) 5mm microunit.

The study began with the removal of temporary implant-supported crown and prosthetic components. Firstly, the type 1 smartpeg was inserted with manual torque of approximately 4 to 6 N.cm directly in the implant platform (G1), in accordance with the manufacturer's instructions (Osstell, Göteborg, Sweden). In all the smartpegs (Fig 1), measurements were taken in four directions: mesial, distal, buccal and lingual.

The value obtained by Osstell^®^ was automatically transformed into ISQ values, ranging from 1 to 100. After measurement, the smartpeg was unscrewed. After this, the microunit type component with a transmucosal height of 1mm (G2) was inserted and received 20N torque applied with a manual ratchet. In the microunit, smartpeg component type A3 was inserted with manual torque of approximately 4 to 6 N.cm and all measurements were taken. After measurement, the smartpeg and the 1 mm microunit were unscrewed.

Subsequently, a microunit with a transmucosal height of 5mm was inserted in the same implant (G3), and received 20N torque applied with a manual ratchet. The smartpeg type A3 was inserted with a manual torque of approximately 4 to 6 N.cm and all measurements were taken. After measurement, the smartpeg and the microunit type component were unscrewed.

The provisional prosthesis were reinserted on the implant; the screw hole was sealed with a polyester strip and the provisional restorative Bioplic (Biodinâmica, Ibiporã, Paraná, Brazil).

### Data analysis

Statistical analysis was performed by the R software (3.2.4 version) with a 5% level of significance. The Kolmogorov-Smirnov test showed non-normal data distribution. The Friedman test was used for inter-group comparisons. The Wilcoxon post-hoc test with Bonferroni correction (p<0.016) were used. The relationship between the ISQ values and measurement distances was verified by Spearman correlation.

To quantify the influence of the distances in the mean level of ISQ, the GEE (Generalized Equations Estimating) [15] model was used. This method is suitable to include the existing correlation between repeated measures. The GEE method is known as Marginal Models and can be considered as an extension of the Generalized Linear Models [16] which directly incorporate the correlation between the measurements of the same sample unit. The use of Marginal models has been preferred as an extension of Generalized Linear Models due to its easy interpretation and lack of probability distribution assumption [17]. The √distance was used as predictor variable in the regression model.

## Results

Twelve participants (05 men, 07 women), mean age 52.83 (± 3.77) years (range from 47 to 60), contributed 31 dental implants to the study. The mean ISQ for G1 was 88.27 (±5.70); G2, 72.75 (±4.73) and G3, 66.33 (±3.67). There was statistical significant difference among groups (Table 1). The abutments were inserted and removed without complication.

**Table 1:** ISQ comparisons among groups.

Group	Mean (SD)	Friedman test	Post-hoc test
Platform (G1)	88.27 (5.70)		G1XG2: <0.001
1mm (G2)	72.75 (4.73)	<0.001	G1XG3: <0.001
5mm (G3)	66.33 (3.67)		G2XG3: <0.001

There was statistically significant negative correlation between the ISQ values and the measurement distance (r_s_:-0.852; p<0.001; R^2^:0.553). The measurement distance was significantly associated (B: 0.88, p<0.001) with ISQ value in the univariate regression analysis (Table 2).

**Table 2:** Log-linear regression of the variable that predicts the ISQ value.

Variable	β	Exp(β)	C.I—95%	P-value
Intercept	4.46	-	-	-
√Distance	-0.13	0.88	[0.87; 0.89]	<0.001

## Discussion

The RFA is the main method used for evaluating implant stability in long-term studies. These studies generally begin with surgical insertion of the implant and evaluations continue through to function of the prosthesis [18]. Ideally, the implant-supported prosthesis requires an abutment because of all the benefits it brings. However, after the abutments have been inserted, their removal is strongly contraindicated, because this breaks the bacterial seal at the implant interface component [19]. Furthermore, abutment removal can damage the threads of the implant, the abutment itself, and even cause loss of the prosthesis [20].

Therefore, long term studies of the behavior of dental implants have performed RFA measurements of both platforms and abutments. However, these values cannot be compared because they are not the same as the values shown in this study.

Because the transducers fixed to components or implant platform (2) print the lateral force on the prosthetic component when the measurement is performed, the displacement is expected to be larger, since it increases the lever arm in relation to the measurement made directly on the platform. Thus, when the ISQ component is measured, a lower value is expected for the same implant, compared with the value measured directly on the platform.

In the present study, two micro unit abutments with the lower and higher transmucosal heights available (1 and 5mm) were selected for each implant, and we observed that the more the transmucosal heights increased, the lower was the ISQ value. Moreover, all the values on the abutments were lower than those on the implant platform, for the same implants.

The abutments selected for the study were of the microunit type, due to the nature of screw-retained prostheses. The sample with external hexagon implants was chosen, because removal of the abutments was worse and less indicated for the Morse cone type. Futures studies are necessary to investigate the behavior of the ISQ values in dental implants with Morse cone connections.

[21] Evaluated the RFA from various aspects, but always took measurements directly on the implant platform. Their study showed that the diameter of the implant and the direction of measurement (parallel or perpendicular) relative to smartpeg did not significantly alter the ISQ value. The only statistically significant correlation found was between insertion torque and ISQ values. [22] Suggested that Osstell^®^ can be used for diagnosis of implant stability and evaluation of circular bone loss. But for partial bone defects they believed that the measurements were inaccurate.

Some articles have reported the comparison between the ISQ measured on the abutment and the value measured directly on the implant platform [23–27]. In the present study, the log-linear marginal regression indicated that each unit increased in the square root of the smartpeg height, tended to decrease about 12% the mean ISQ value. The findings of this study suggest that the ISQ values obtained on the abutment cannot be compared with the platform values. This statement must be justified by the fact that the abutment has a transmucosal height, and consequently the force would be applied at a distance from the implant platform. As regards this result, the higher the abutment height, the lower the ISQ tended to be. Therefore, the ISQ values measured directly on prosthetic abutments in longitudinal studies were lower than the baseline ISQ values. These comparisons of ISQ results should be considered tilted. The authors of the present study suggest that to report and compare ISQ, studies should do this in a standardized way, always measured in the same place (either on platforms or abutments).

A scale has been established that correlates the ISQ value with the stability of the implant, determining ranges of values as a prognosis for success of the implant. However, the results obtained in this study, demonstrated that the ISQ varies relative to where this measurement is made. For the scale values, it should be clear where the measurement was made, and if measured on different abutments, it must be stated which the benchmarks were. Another methodological implication is that as the abutment transmucosal height increases, a lower ISQ value is expected, which may not reach the value of 100 proposed as the maximum value of RFA [28,29]. Consequently, a lower ISQ value can be considered ideal and/or maximum according to the component on which it was measured.

The RFA (by means of ISQ) is more sensitive for detecting changes in implant stability than the conventional clinical and radiological examinations. It is possible to detect the loss of stability before clinical signs/symptoms including pain and mobility. When the problem is diagnosed in time, the adoption of prudent measures could revert the drop in RF values [30,31]. Given the clinical importance and the present results, it is important to standardize the measurement place (or equivalence between measurements) of ISQ values in order to allow longitudinal monitoring of the implant, and not to make misleading comparisons (e.g., measurement on the platform at baseline compared with measurement on the abutment at follow-up), leading to false diagnoses and consequently an under- or over-treatment.

Thus, the relationship between RFA values and the implant platform and the prosthetic components may represent a huge advance in longitudinal research in implantology.

This study may present limitations, such as the use of only external hexagon implants. The authors suggest that further studies must be conducted with different diameters and implant connection, and different components to confirm the present findings, or not. They also suggests that studies should be developed to establish a mathematical formula for equating the ISQ value measured on the platform and at different transmucosal abutment heights. New scales of values for each ISQ and transmucosal abutment height should be further investigated.

## Conclusion

The abutment height had a significant impact on resonance frequency analysis measurements. The higher the transmucosal abutment height, the lower was the implant stability quotient value.

In clinical practice, it is suggested that the ISQ measured on the abutment cannot be compared with values measured on the implant platform.

## Supporting information

S1 Table ISQ comparisons among groups. (DOCX)

S2 Table Log-linear regression of the variable that predicts the ISQ value. (DOCX)
