# Supplementary material for: Relationship between implant stability on the abutment and platform level by means of resonance frequency analysis: A cross-sectional study
Source: PLoS One. 2017 Jul 24;12(7):e0181873. doi: 10.1371/journal.pone.0181873 (PMC5526494; doi:10.1371/journal.pone.0181873)
Supplement: S1 Table — (DOCX) [file pone.0181873.s001.docx]

| Table 1. ISQ comparisons among groups. | | | |
| --- | --- | --- | --- |
| Group | Mean (SD) | Friedman test | Post-hoc test |
| Platform (G1) | 88.27 (5.70) |  | G1XG2: <0.001 |
| 1mm (G2) | 72.75 (4.73) | <0.001 | G1XG3: <0.001 |
| 5mm (G3) | 66.33 (3.67) |  | G2XG3: <0.001 |
|  |  |  |  |
