# Supplementary material for: Relationship between implant stability on the abutment and platform level by means of resonance frequency analysis: A cross-sectional study
Source: PLoS One. 2017 Jul 24;12(7):e0181873. doi: 10.1371/journal.pone.0181873 (PMC5526494; doi:10.1371/journal.pone.0181873)
Supplement: S2 Table — (DOCX) [file pone.0181873.s002.docx]

| **Table 2.** Log-linear regression of the variable that predicts the ISQ value**.** | | | | |
| --- | --- | --- | --- | --- |
| **Variable** | **β** | **Exp(β)** | **C.I - 95%** | **P-value** |
| Intercept | 4.46 | - | - | - |
| √Distance | -0.13 | 0.88 | [0.87; 0.89] | <0.001 |
